# Supplementary material for: A Global Screen for Assembly State Changes of the Mitotic Proteome by SEC-SWATH-MS
Source: Cell Syst. 2020 Feb 26;10(2):133–155.e6. doi: 10.1016/j.cels.2020.01.001 (PMC7042714; doi:10.1016/j.cels.2020.01.001)

Q8IXH7 | NELFD_HUMAN | NELFCD NELFD TH1 TH1L HSPC130
Monomer MW [kDa]: 66.247 Monomer expected elution fraction: 44

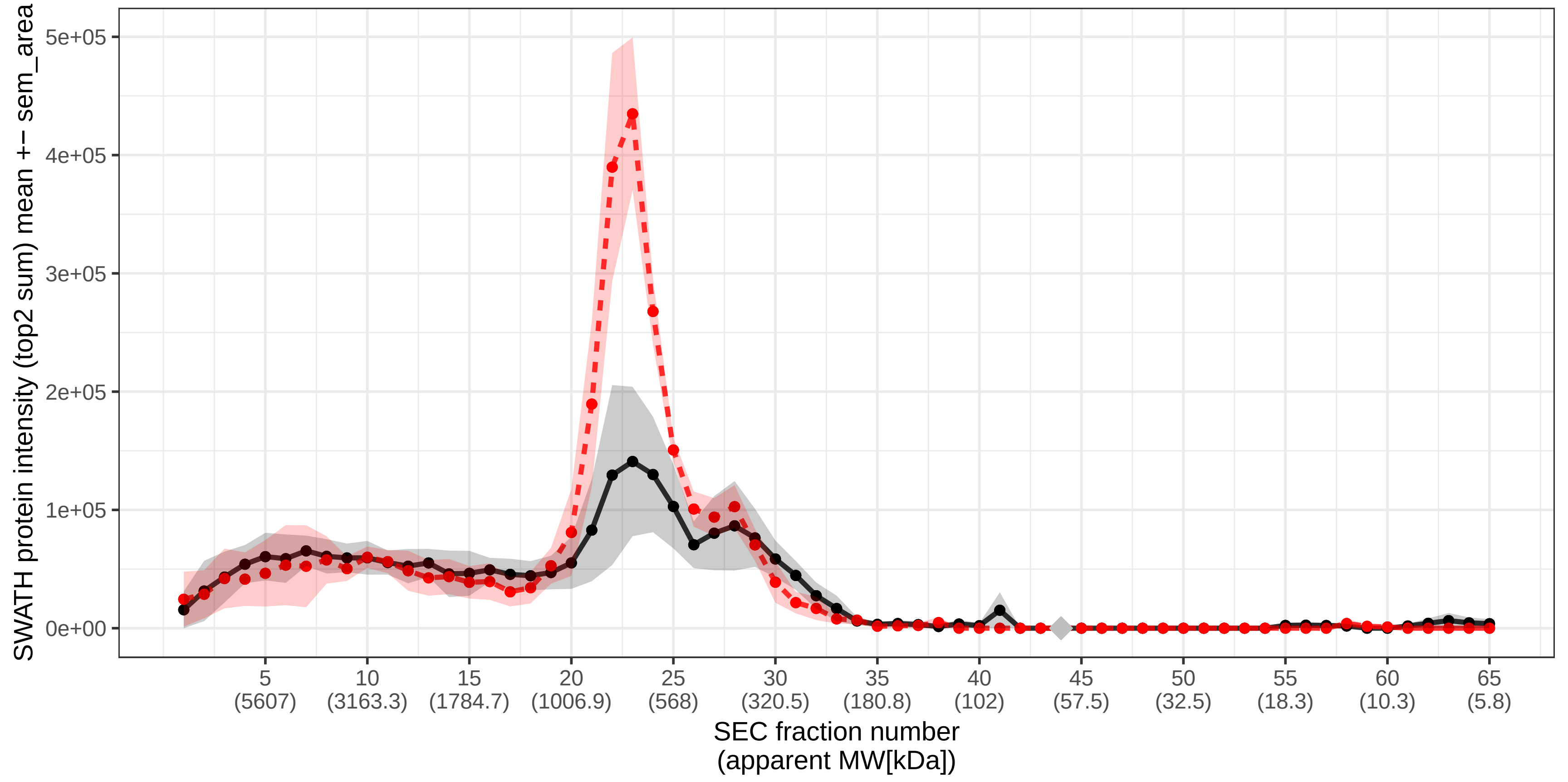

Supplement: Data S1. SEC-SWATH-MS Protein Chromatograms, Related to Figure 1 [file mmc6.zip › SECchrom_Q8IXH7_NELFD_HUMAN_NELFCD_NELFD_TH1_TH1L_HSPC130.pdf]
